# Supplementary material for: Field-scale crop water consumption estimates reveal potential water savings in California agriculture
Source: Nat Commun. 2024 Mar 25;15:2366. doi: 10.1038/s41467-024-46031-2 (PMC10963747; doi:10.1038/s41467-024-46031-2)
Supplement: Supplementary file 1 — Supplementary Information [file 41467_2024_46031_MOESM1_ESM.pdf]

Field-scale crop water consumption estimates reveal potential water savings in California agriculture

Supplementary Information

Anna Boser[1*], Kelly Caylor[1,2], Ashley Larsen[1],
Madeleine Pascolini-Campbell[3], John T. Reager[3], Tamma Carleton[1,4]

[1*]Bren School of Environmental Science and Management, UC Santa Barbara, 2400 Bren Hall, Santa Barbara, 93106, CA, USA.
[2]Department of Geography, UC Santa Barbara, Ellison Hall, Santa Barbara, 93106, CA, USA.
[3]NASA Jet Propulsion Laboratory, California Institute of Technology, 4800 Oak Grove Drive, Pasadena, 91109, CA, USA.
[4]National Bureau of Economic Research, 1050 Massachusetts Avenue, Cambridge, 02138, MA, USA.

*Corresponding author(s). E-mail(s): annaboser@ucsb.edu;
Contributing authors: caylor@ucsb.edu; larsen@bren.ucsb.edu;
madeleine.a.pascolini-campbell@jpl.nasa.gov; john.reager@jpl.nasa.gov;
tcarleton@ucsb.edu;

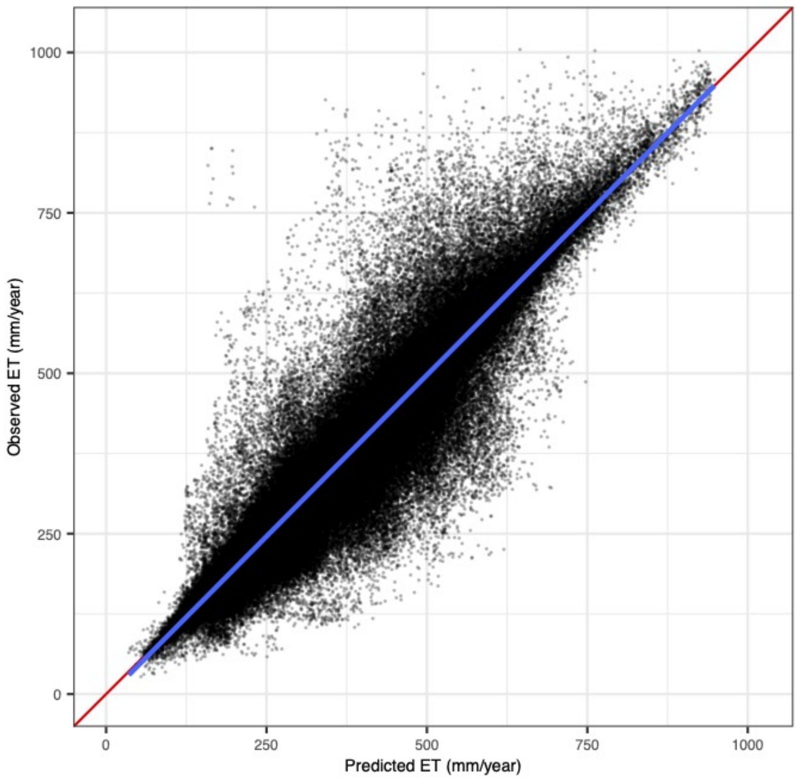

Supplementary Fig. 1 Observed versus predicted naturally-occurring evapotranspiration (ET) over fallow lands. Each point represents one 70m pixel of purely fallow land during 2016, 2018, or 2019. The model is unbiased in its predictions and achieves an R^2 of 0.87 and a MAE of 36 mm per year.

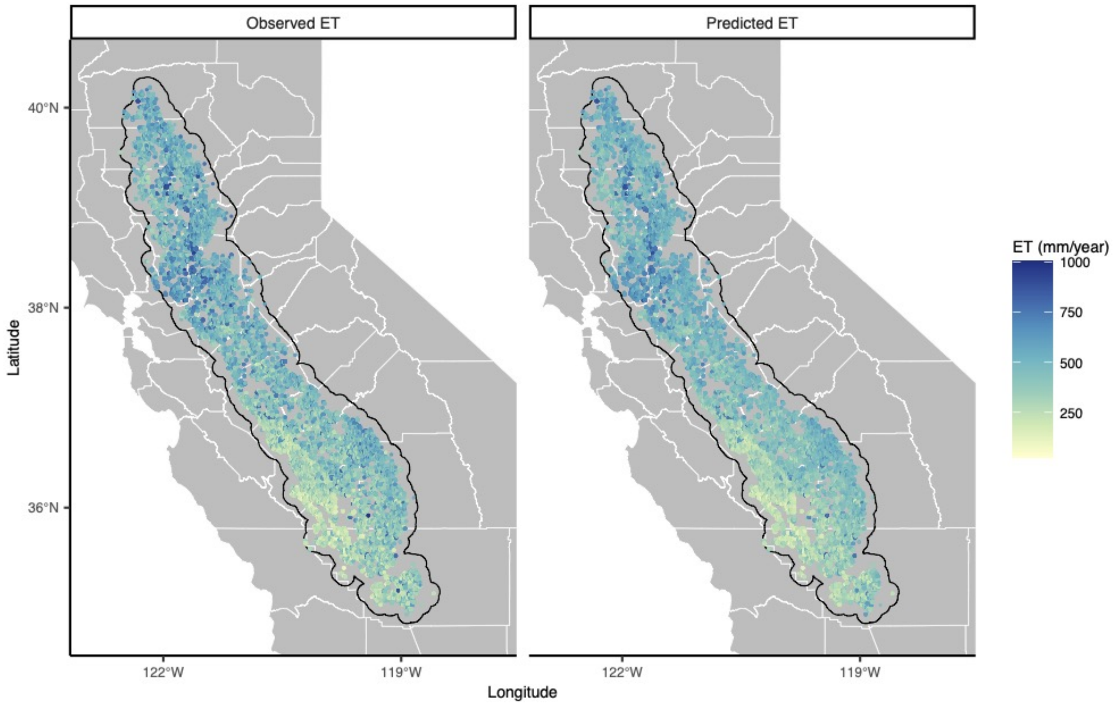

Supplementary Fig. 2 Observed versus predicted naturally-occurring evapotranspiration (ET) over fallow fields in the California Central Valley. The model is able to accurately reconstitute naturally-occurring ET in all regions represented in the test set.

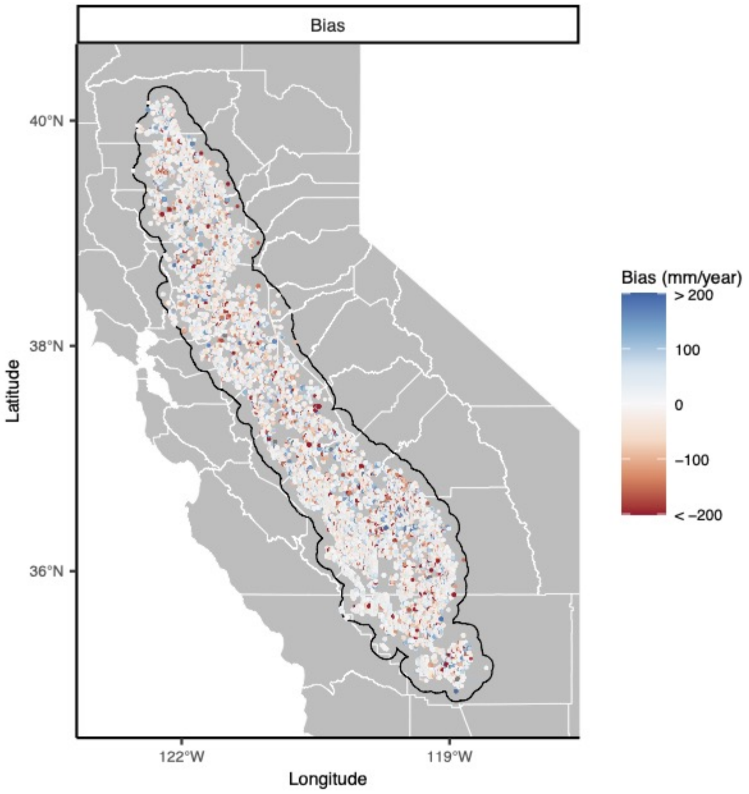

Supplementary Fig. 3 Spatial error structure of naturally-occurring evapotranspiration (ET) predictions over fallow lands. Error, or bias, is calculated by taking the difference between the predicted ET and observed ET. There appears to be no spatial structure to the bias. In other words, the model is not more likely to be positively or negatively biased based on the region it is predicting over.

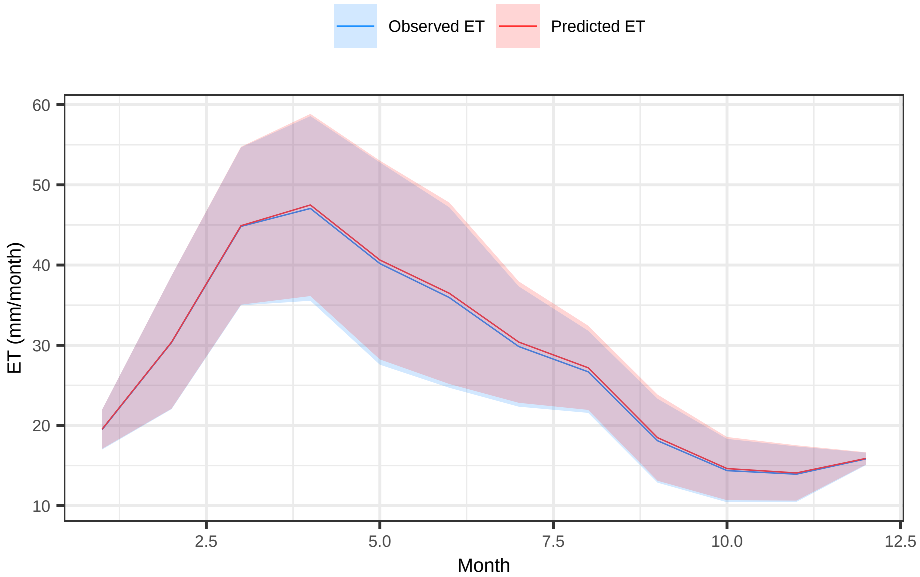

Supplementary Fig. 4 Temporal error structure of naturally-occurring evapotranspiration (ET) predictions over fallow lands. The average ET observed and predicted over fallow lands are similar throughout the year. In other words, the model is not more likely to be positively or negatively biased based on the month it is predicting over.

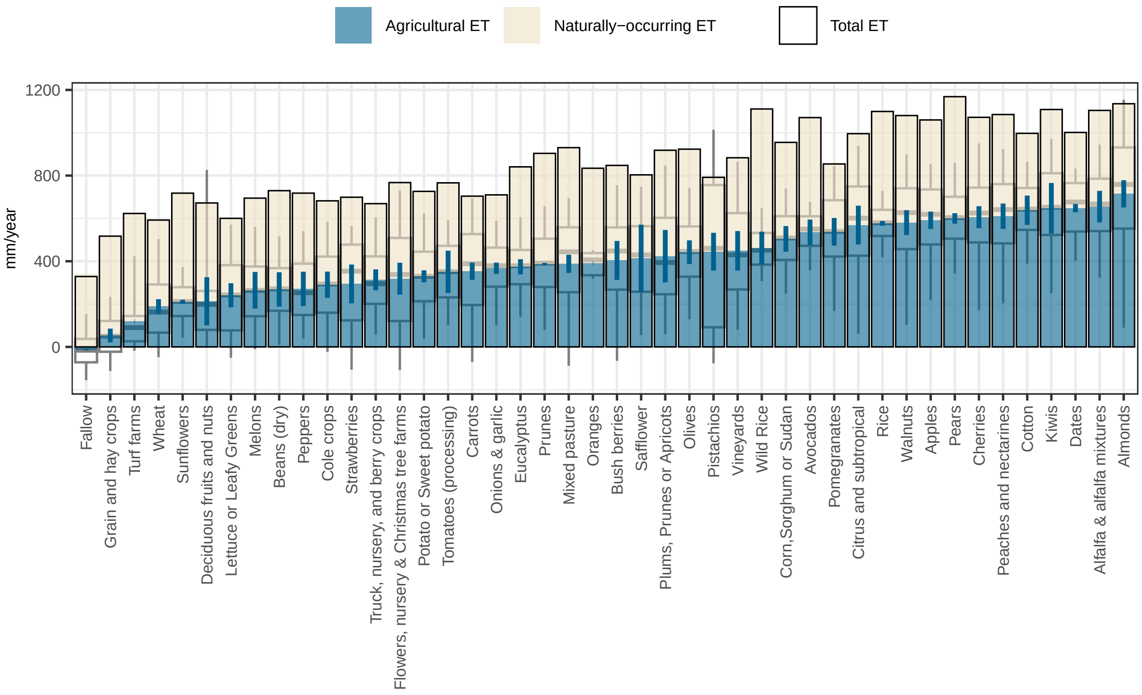

Supplementary Fig. 5 Observed, fallow, and agricultural evapotranspiration (ET) by crop type in the Central Valley. Mean agricultural ET by crop type (blue fill and 95% CI) is the average difference between observed ET (black outline) and naturally-occurring ET (cream fill). While we find significant differences in mean agricultural ET across crop types, the gray box plots also show a broad spread in agricultural ET within crop types (box plot shows 0.5, 0.25. 0.5, 0.75, and 0.95 quantiles).

Supplementary Note 1: Empirical vs. modeled agricultural ET

In the absence of empirical agricultural evapotranspiration (ET) estimates, modeling theoretical crop water demand can be a useful tool for water management. One popular model developed specifically for California is CalSIMETAW, a model that estimates crop-specific water demand at the county level using a combination of crop coefficients which are a measure of crop-specific water intensity, weather data, and several other variables [1].

County-level, crop-specific water demand estimates calculated using CalSIMETAW are available for years 2000-2015. To retrieve modeled estimates of agricultural ET from CalSIMETAW's water demand estimates, we try removing naturally-occurring ET in two different ways: (1) by subtracting our empirical estimates of naturally-occurring ET or (2) by subtracting precipitation as reported by CalSIMETAW. In line with how agricultural ET is generally calculated using modeling, we calculate agricultural ET at a monthly time step and set it to zero if it is negative before aggregating to a yearly estimate. To compare these to our estimates of agricultural ET, we aggregate our empirical agricultural ET estimates to corresponding crop categories at the county level, also computed at the monthly level using the protocol just described.

We find a correlation of 0.74 between empirical and modeled county-level, crop-specific agricultural ET when using our naturally-occurring ET estimates as the counterfactual for the modeled estimates (Supplementary Fig. 6A). Modeled agricultural ET has a positive bias of 14.1 cm per year (12.4-15.8 95% CI) as compared to the empirical measurements. Using precipitation as the counterfactual deteriorates the relationship between empirical and modeled agricultural ET: the correlation drops to 0.69 and the bias increases to 34.2 cm per year (32.1-36.3 95% CI) (Supplementary Fig. 6B).

We note the discrepancy in years used to calculate the modeled (2000-2015) versus empirical (2016, 2018, 2019) agricultural ET estimates. However, we believe that the biases found are not a result of this difference: if the climate is different between these sets of years, the more recent years are those that have been most hot and dry [2]. As a result, one could expect that the bias to be even larger if the same years were used for the analysis.

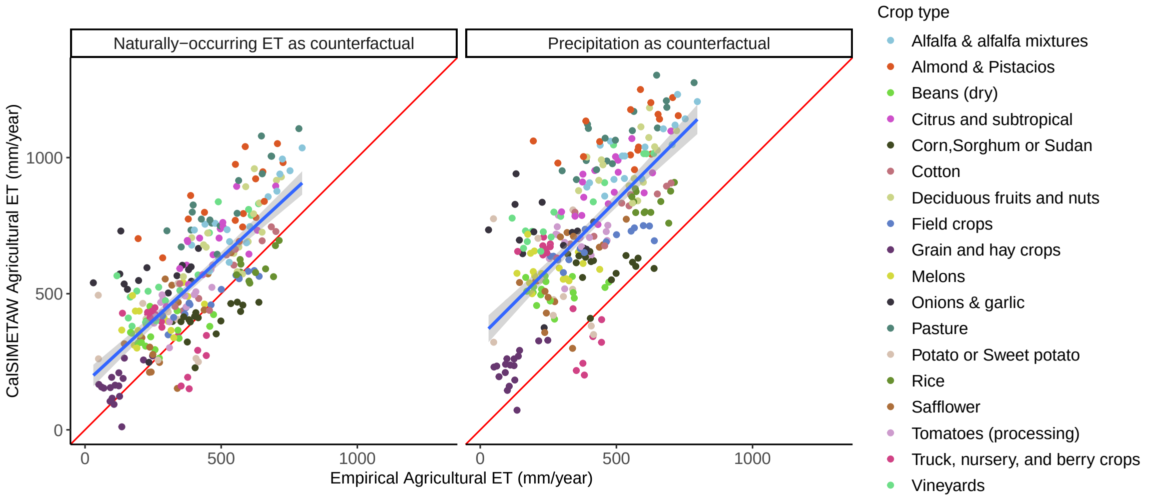

Supplementary Fig. 6 Simulated vs. empirical agricultural evapotranspiration (ET). Simulated agricultural ET is calculated using theoretical crop water demand from the CalSIMETAW model and by subtracting either (A) naturally-occurring ET estimated using machine learning or (B) precipitation. Each point represents the agricultural ET for a crop in a specific county in the California Central Valley.

Supplementary Note 2: The effect of orchard age on agricultural ET

Water consumption is known to be affected by orchard age, especially for young orchards [3]. One advantage of data-driven, field-scale estimates of agricultural ET is that they can capture such variation.

Since information about orchard age is not currently available in California, we use the LandIQ dataset's transitions from "young perennial" classifications to crop-specific orchard categories as a proxy for orchard age. "Young perennials" refer to non-fruit-bearing young trees, so their change to fruit-bearing orchard classifications helps us estimate the age of the orchard. LandIQ crop type data are available for the years 2014, 2016, 2018, 2019, and 2020. Since we have agricultural ET data for the years 2016, 2018, and 2019, we can date orchards that have been bearing fruit for up to five years (i.e. these would be orchards for which we have agricultural ET data in 2019, and were classified as young perennials for the last time in 2014.) Using this technique, we uncover a clear relationship between orchard age and agricultural ET (Supplementary Fig. 7).

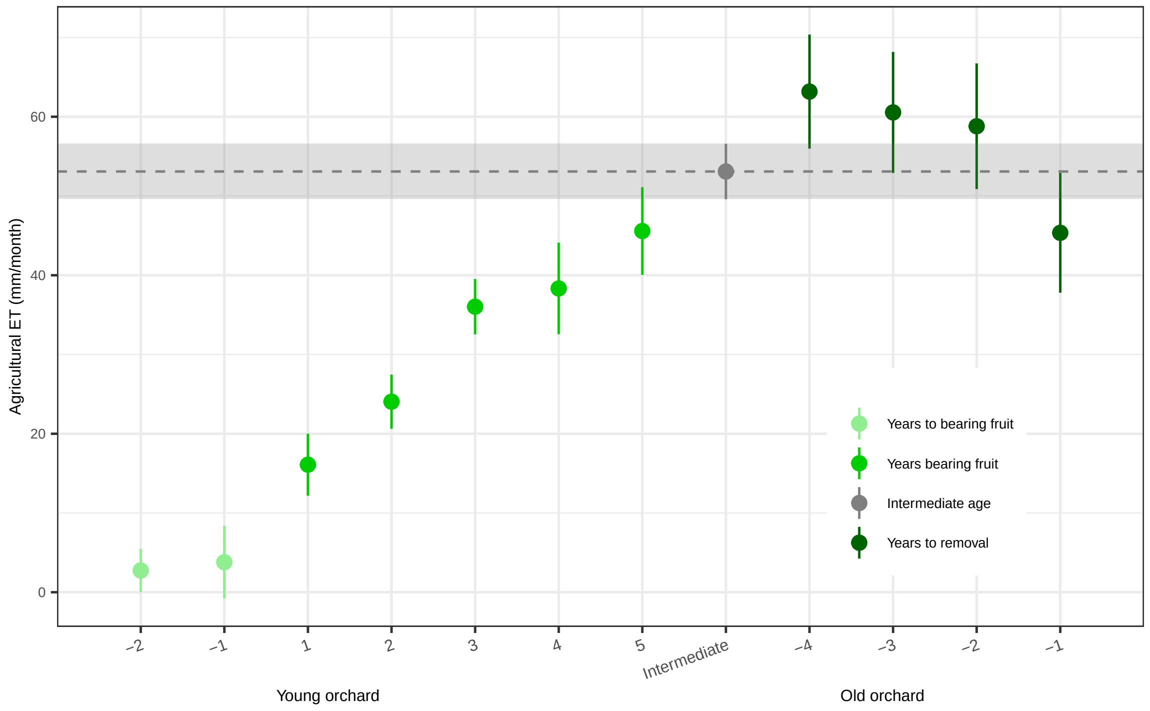

Supplementary Fig. 7 Water consumption by life stage of orchards in California's Central Valley. Prior to bearing fruit, orchards have low agricultural water consumption, or evapotranspiration (ET). Water consumption then increases rapidly during the first 5 years of fruit production, after which it stabilizes.

Supplementary Note 3: Empirical vs. theoretical irrigation efficiency

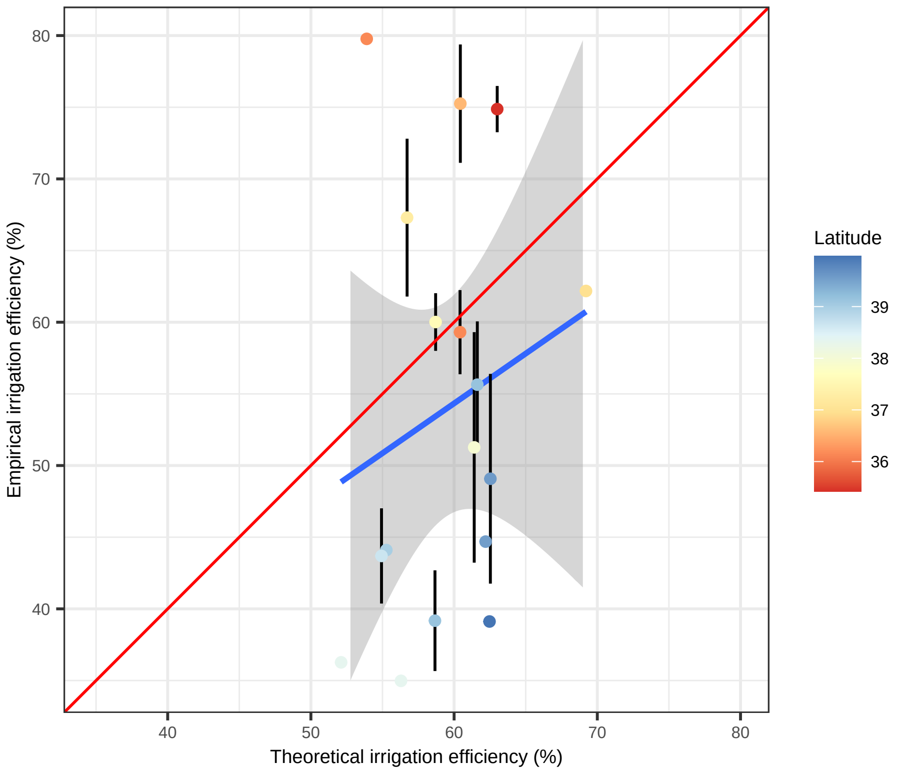

Supplementary Fig. 8 Empirical vs. theoretical irrigation efficiency across the counties of the California Central Valley. Empirical irrigation efficiency is calculated using empirical estimates of agricultural ET divided by reports of total water diverted for irrigation. Theoretical irrigation efficiency is calculated based on efficiency estimates and reported irrigation technology used in each county. Theoretical irrigation efficiency underestimates empirical estimates for more southern counties and overestimates it for more northern counties.

In the absence of empirical irrigation efficiency measures, irrigation efficiency is often approximated [4, 5] using technology-specific irrigation efficiency estimates drawn from reports [6–8]. Specifically, irrigation efficiency is calculated as the product conveyance efficiency, management efficiency, and application efficiency. Conveyance efficiency captures losses bringing water from its source to the farm, management efficiency describes losses on the farm, and application efficiency captures losses during or after application. We calculate county-specific irrigation efficiency by pulling efficiency numbers from the aforementioned reports. We set conveyance efficiency to .85, management efficiency to .95, and application efficiency to .60 for flood irrigation, .75 for sprinkler irrigation, and .95 for drip irrigation. The frequency of adoption of different irrigation technologies in each county is retrieved from the same USGS dataset we obtain reports of water diverted for irrigation from.

Our empirical estimates of irrigation efficiency are not significantly different to what theoretical estimates would predict, though theoretical estimates tend to underestimate efficiency in southern counties and overestimate it in northern counties (Supplementary Fig. 8). It is possible that theoretical numbers underestimate the irrigation efficiency in the south because they only capture improvements in irrigation technology, and not any other practices that might increase efficiency. For example, deficit irrigation can improve irrigation efficiency [9], as well as careful irrigation scheduling [10] and precision agriculture [11]. Empirical estimates of irrigation efficiency may therefore capture the effect of additional drivers of irrigation efficiency past the technology being used.

Supplementary Note 4: The effect of OpenET and machine learning model error on agricultural ET estimates

We define agricultural ET as the difference between total ET and naturally-occurring ET, $\mathrm{ET}_{\mathrm{ag}} = \mathrm{ET}_{\mathrm{tot}} - \mathrm{ET}_{\mathrm{nat}}$ (Main text eq. 1), where naturally-occurring ET is the counterfactual ET that would occur naturally, were the same land fallow. However, we calculate agricultural ET using estimates of total ET from OpenET and modeled naturally-occurring ET trained on OpenET data (Supplementary eq. 1). Since the OpenET data and the model we use to estimate naturally-occurring ET have errors associated with them, our estimates of agricultural ET can be described as the true agricultural ET plus several error terms (Supplementary eq. 4), which we derive as follows:

$$\mathrm{ET}^{*}_{\mathrm{ag}} = \hat{\mathrm{ET}}_{\mathrm{tot}} - \tilde{\hat{\mathrm{ET}}}_{\mathrm{nat}} \tag{1}$$

where $\mathrm{ET}^{*}_{\mathrm{ag}}$ denotes our estimate of agricultural ET, $\hat{\mathrm{ET}}_{\mathrm{tot}}$ is OpenET's estimate of $\mathrm{ET}_{\mathrm{tot}}$ and $\tilde{\hat{\mathrm{ET}}}_{\mathrm{nat}}$ is our estiate of the naturally-occurring ET, predicted by our machine learning (ML) model. The hat notation indicates estimates inclusive of OpenET error, while the tilde indicates estimates inclusive of ML model error.

Because our naturally-occurring ET ML model is trained on and therefore predicts OpenET values over fallow lands, we can rewrite the same expression as:

$$\mathrm{ET}^{*}_{\mathrm{ag}} = \hat{\mathrm{ET}}_{\mathrm{tot}} - (\hat{\mathrm{ET}}_{\mathrm{nat}} + \epsilon_{\mathrm{ML}}) \tag{2}$$

where $\hat{\mathrm{ET}}_{\mathrm{nat}}$ is the (naturally-occurring) ET that OpenET would observe if the field were fallow, and ϵ_{ML} is the error from the ML model in predicting $\hat{\mathrm{ET}}_{\mathrm{nat}}$.

We can then again reorganize the same equation by separating the error from the OpenET observations. This reveals the relationship between the true ET_{ag} and our estimate, $\mathrm{ET}^{*}_{\mathrm{ag}}$:

$$\mathrm{ET}^{*}_{\mathrm{ag}} = \mathrm{ET}_{\mathrm{tot}} + \epsilon_{\mathrm{OpenET,ag}} - (\mathrm{ET}_{\mathrm{nat}} + \epsilon_{\mathrm{OpenET,fal}} + \epsilon_{\mathrm{ML}}) \tag{3}$$

$$= \mathrm{ET}_{\mathrm{ag}} + \epsilon_{\mathrm{OpenET,ag}} - \epsilon_{\mathrm{OpenET,fal}} - \epsilon_{\mathrm{ML}} \tag{4}$$

where ET_{ag}, $\mathrm{ET}_{\mathrm{tot}}$, and $\mathrm{ET}_{\mathrm{nat}}$ are the true (unobservable) agricultural ET, total ET, and naturally-occurring ET, respectively, $\epsilon_{\mathrm{OpenET,ag}}$ is the error associated with the OpenET estimate over the agricultural field and $\epsilon_{\mathrm{OpenET,fal}}$ is the error associated with what the OpenET estimate would be if the land were fallow.

In our study, we seek to estimate mean $\mathrm{ET}_{\mathrm{ag}}$, be it over the entire Central Valley, for a certain crop type, or for a given county, we seek to calculate the expectation of $\mathrm{ET}_{\mathrm{ag}}$ over a sample of grid cells. Using Supplementary eq. 5, we can show that our estimate of $\mathbb{E}[\mathrm{ET}^{*}_{\mathrm{ag}}]$ depends not only on $\mathbb{E}[\mathrm{ET}_{\mathrm{ag}}]$, but also the expectation of the error terms.

$$\mathbb{E}[\mathrm{ET}^{*}_{\mathrm{ag}}] = \mathbb{E}[\mathrm{ET}_{\mathrm{ag}}] + \mathbb{E}[\epsilon_{\mathrm{OpenET,ag}}] - \mathbb{E}[\epsilon_{\mathrm{OpenET,fal}}] - \mathbb{E}[\epsilon_{\mathrm{ML}}] \tag{5}$$

We show in Supplementary Figures 1-4 that ϵ_{ML} is unbiased, that is, $\mathbb{E}[\epsilon_{\mathrm{ML}}] = 0$. OpenET has also been shown to produce unbiased estimates over agricultural lands, so $\mathbb{E}[\epsilon_{\mathrm{OpenET,ag}}] = 0$ (see Table 3 on page 6 of the OpenET Intercomparison and Accuracy report) [12]. We can also assume $\epsilon_{\mathrm{OpenET,fal}}$ is unbiased because though OpenET has not specifically been evaluated over fallow fields, it produces unbiased estimates over natural shrublands and grasslands with ET values of similar magnitude to fallow fields in the Central Valley [12]. As a result, in expectation our estimates of agricultural ET are unbiased, and consequently produce unbiased regression coefficients.

$$\mathbb{E}[\mathrm{ET}^{*}_{\mathrm{ag}}] = \mathbb{E}[\mathrm{ET}_{\mathrm{ag}}] \tag{6}$$

While our estimates of agricultural ET are unbiased in expectation, the error terms do add variance that is not present in the true agricultural ET. Therefore, any analysis that assesses variation

across pixels will reflect inflated variance relative to true agricultural ET. For example, this has implications for the farming practices and fallowing scenarios we conduct (Fig. 3). However, here we estimate that error is responsible for only 11% of the variance in our annual estimates of agricultural ET, suggesting its influence over key results is limited. Specifically, if we assume that all error terms are independent, we have:

$$\mathrm{var}(\mathrm{ET}^{*}_{\mathrm{ag}}) = \mathrm{var}(\mathrm{ET}_{\mathrm{ag}}) + \mathrm{var}(\epsilon_{\mathrm{OpenET,ag}}) + \mathrm{var}(\epsilon_{\mathrm{OpenET,fal}}) + \mathrm{var}(\epsilon_{\mathrm{ML}}) \tag{7}$$

where $\mathrm{var}(\cdot)$ denotes the variance.

Then, because variance is equal to mean squared error (MSE) when bias is 0, we have:

$$\mathrm{var}(\mathrm{ET}^{*}_{\mathrm{ag}}) = \mathrm{var}(\mathrm{ET}_{\mathrm{ag}}) + \mathrm{MSE}(\epsilon_{\mathrm{OpenET,ag}}) + \mathrm{MSE}(\epsilon_{\mathrm{OpenET,fal}}) + \mathrm{MSE}(\epsilon_{\mathrm{ML}}) \tag{8}$$

We calculate the MSE of our machine learning model using our test set, and OpenET provides root MSE values over croplands and shrublands (which we use to approximate error over fallow lands) (again, see Table 3 on page 6 of the OpenET Intercomparison and Accuracy report) [12]. We find that for estimates of yearly $\mathrm{ET}^{*}_{\mathrm{ag}}$, $\mathrm{MSE}(\epsilon_{\mathrm{OpenET,ag}}) + \mathrm{MSE}(\epsilon_{\mathrm{OpenET,fal}}) + \mathrm{MSE}(\epsilon_{\mathrm{ML}})$ represents only 11% of $\mathrm{var}(\mathrm{ET}^{*}_{\mathrm{ag}})$. This indicates that the vast majority of the variance we uncover is representative of true differences in water consumption across fields, as opposed to variability due to model errors.

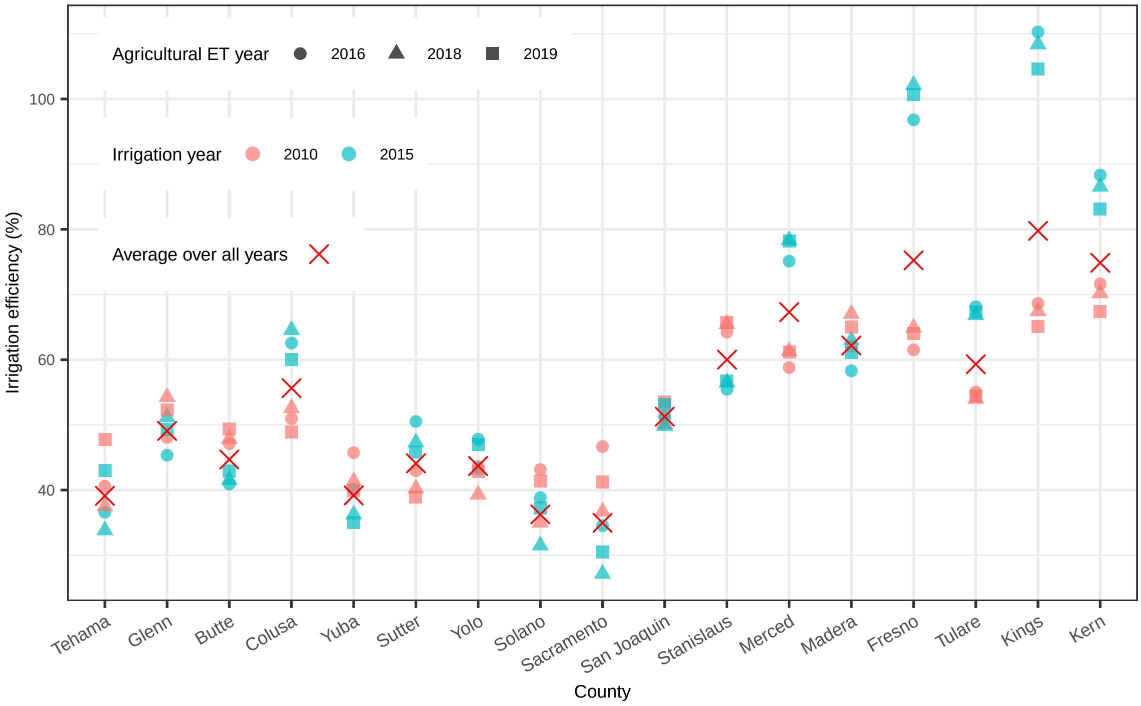

Supplementary Fig. 9 Uncertainty in irrigation efficiency estimates. To calculate irrigation efficiency, we divide agricultural evapotranspiration (ET) by irrigation amount, but the available data do not overlap in years over which they are available. Counties are ordered by latitude, with more northern counties on the left. The average irrigation efficiency is shown as a red x, but we also assess the effect of using different years in the numerator and denominator by combining all different permutations of agricultural ET (shape) and irrigation (color) data. We note that 2016 and 2015 were drought years, while 2018, 2019, and 2010 were wetter. We do find substantial spreads in irrigation efficiency driven by the year of irrigation data, especially in some southern counties. However, most of the spread is driven from combining wet and dry years together, suggesting that an average may successfully decrease much of the variability.

Supplementary Note 5: Results without cleaning the training dataset for the naturally-occurring ET model

When training the naturally-occurring ET model, we remove pixels that are within the top 5% ET during July-September due to some implausibly high numbers during these months that suggest mislabeling of an active agricultural pixel as fallow. Here, we evaluate the effect of this step on our final results.

As one might expect, the agricultural ET estimates recovered without data cleaning are slightly lower as opposed to the numbers reported in the main text (Supplementary Fig. 10). Additionally, the naturally-occurring ET estimates are more erratic across crop groups (Supplementary Fig. 10). Nevertheless, the general trends and conclusions from the main text still hold.

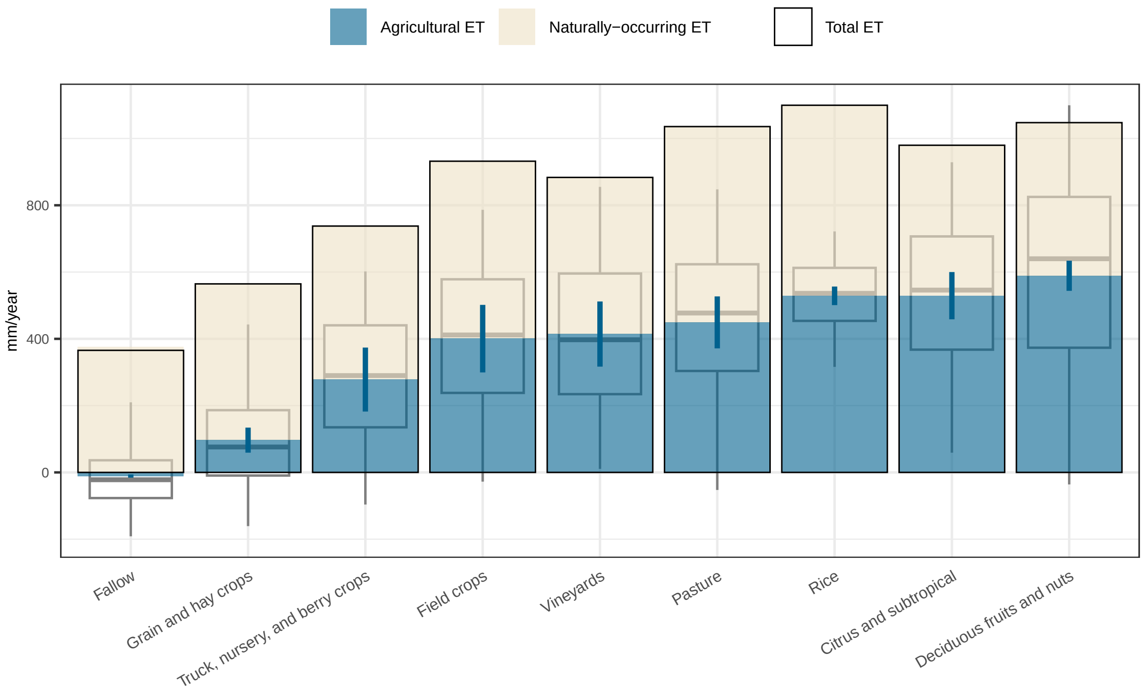

Supplementary Fig. 10 Variations in agricultural evapotranspiration (ET) across and within crop groups without cleaning training data. This is the equivalent of Fig. 2 in the main text, but calculated using a naturally-occurring ET model trained on uncleaned data.

When conducting scenarios, we find that all scenarios are slightly more effective: The crop-switching scenario yields 11.7% savings (95.3% if switching to the minimum-consuming crop), farming practices lead to 13.5% savings, and fallowing 5% of land reduces agricultural ET by 10.0% (Supplementary Fig. 11). All trends are the same as in the main text.

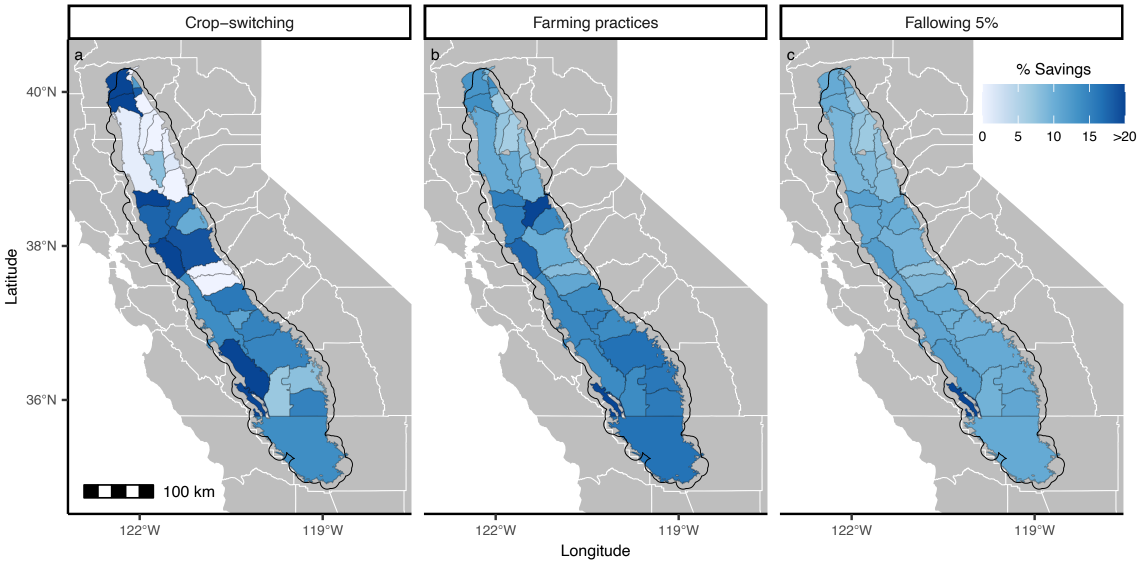

Supplementary Fig. 11 The percent reduction in agricultural evapotranspiration (ET) driven by various management scenarios without cleaning training data. This is the equivalent of Fig. 3 in the main text, but calculated using a naturally-occurring ET model trained on uncleaned data.

As a result of retrieving lower agricultural ET numbers, irrigation efficiency is also generally lower, at 54.6% (46.1, 63.2) on average. However, we still find significantly lower irrigation efficiency in the northern counties (Supplementary Fig. 12).

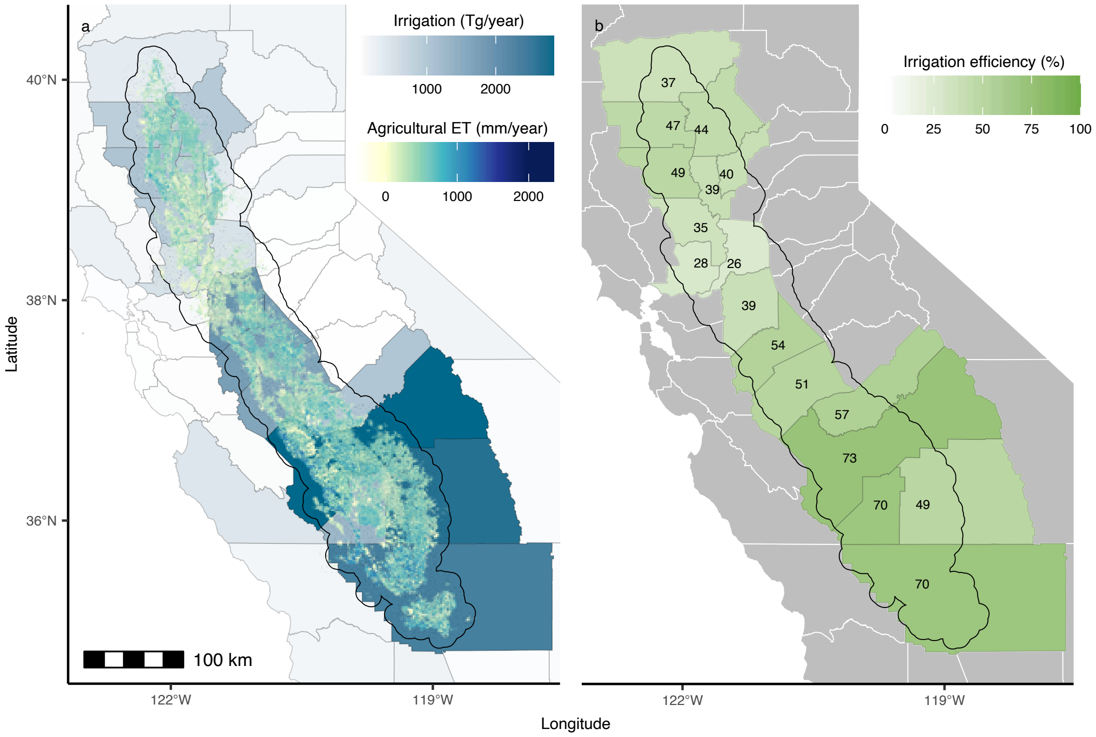

Supplementary Fig. 12 Irrigation efficiency across the counties of the Central Valley without cleaning training data. This is the equivalent of Fig. 4 in the main text, but calculated using a naturally-occurring ET model trained on uncleaned data.

Supplementary Note 6: Results when using training data also marked fallow by the Cropland Data Layer (CDL)

When training the naturally-occurring ET model, we rely on the California Department of Water Resources (DWR) LandIQ crop type dataset due to its high accuracy. Here, we evaluate the effect of instead training the data only on fallow fields that are marked as fallow by both the DWR dataset and the Cropland Data Layer (CDL). Overall, this change does not affect our estimates of naturally-occurring ET: while we estimate 438 mm per year (394.4-481.7 95% CI) in the main text, we estimate 423.9 mm per year (369-478.7 95% CI) here. All general trends and conclusions are robust to this change (Supplementary Fig. 13, Fig. 14, Fig. 15).

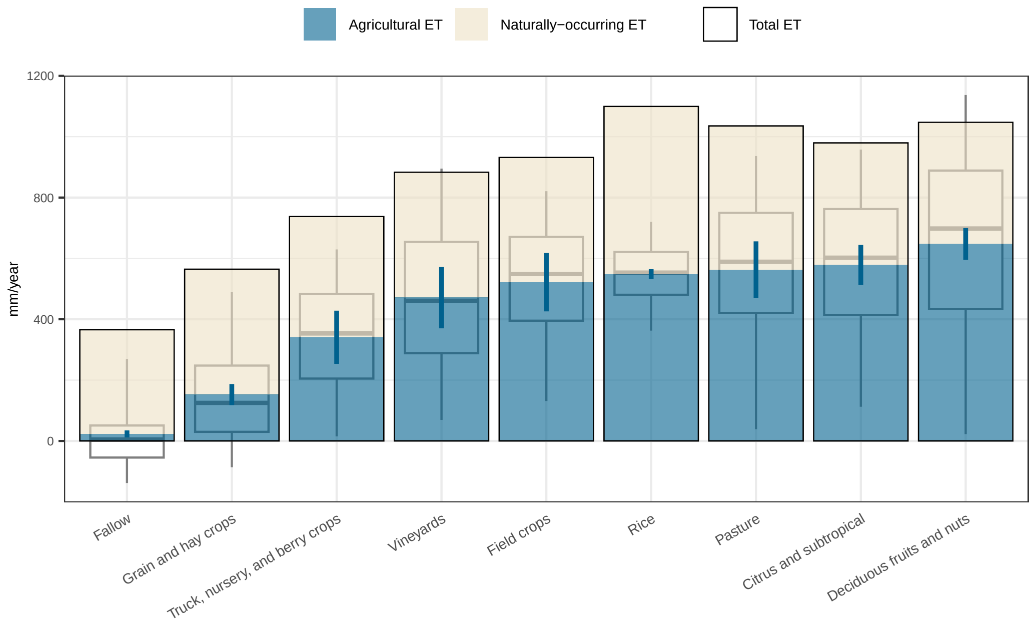

Supplementary Fig. 13 Variations in agricultural evapotranspiration (ET) across and within crop groups using training data also marked fallow by the Cropland Data Layer. This is the equivalent of Fig. 2 in the main text, but calculated using a naturally-occurring ET model trained on data also marked fallow by the Cropland Data Layer.

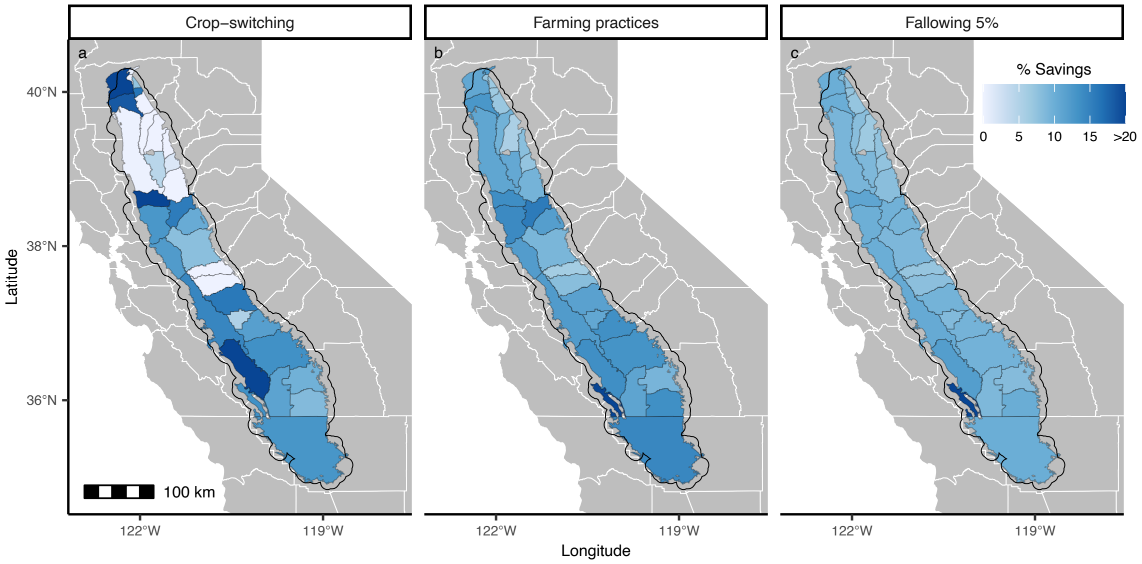

Supplementary Fig. 14 The percent reduction in agricultural evapotranspiration (ET) driven by various management scenarios using training data also marked fallow by the Cropland Data Layer. This is the equivalent of Fig. 3 in the main text, but calculated using a naturally-occurring ET model trained on data also marked fallow by the Cropland Data Layer.

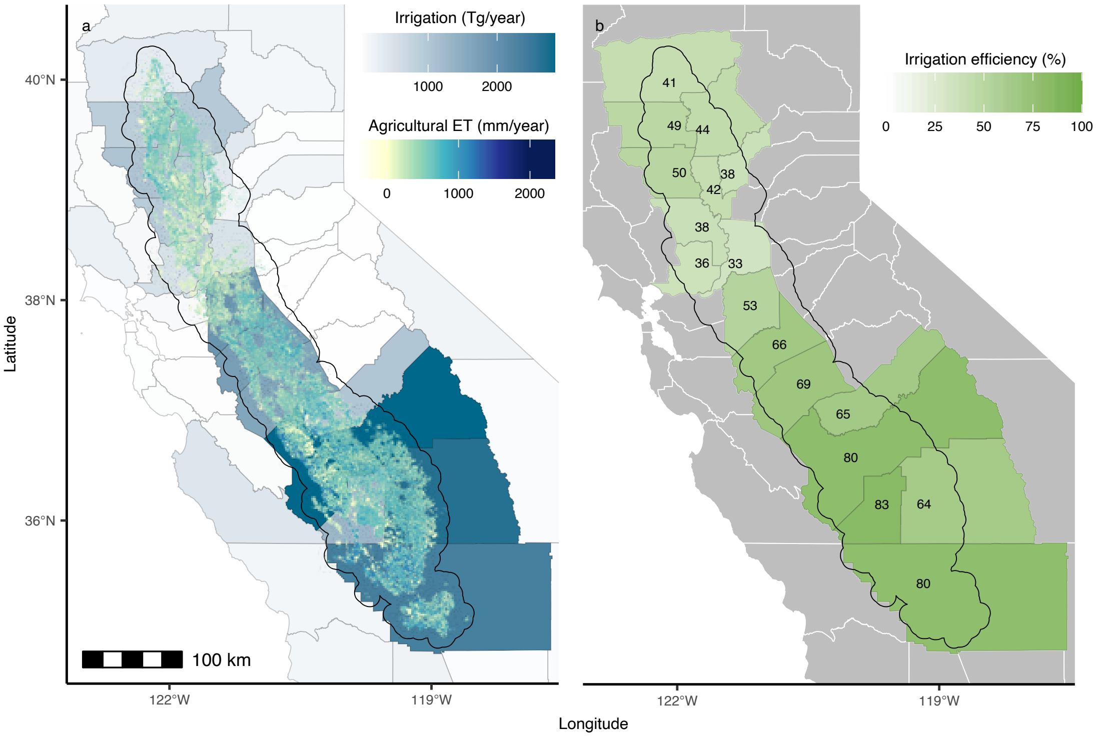

Supplementary Fig. 15 Irrigation efficiency across the counties of the Central Valley using training data also marked fallow by the Cropland Data Layer. This is the equivalent of Fig. 4 in the main text, but calculated using a naturally-occurring ET model trained on data also marked fallow by the Cropland Data Layer.

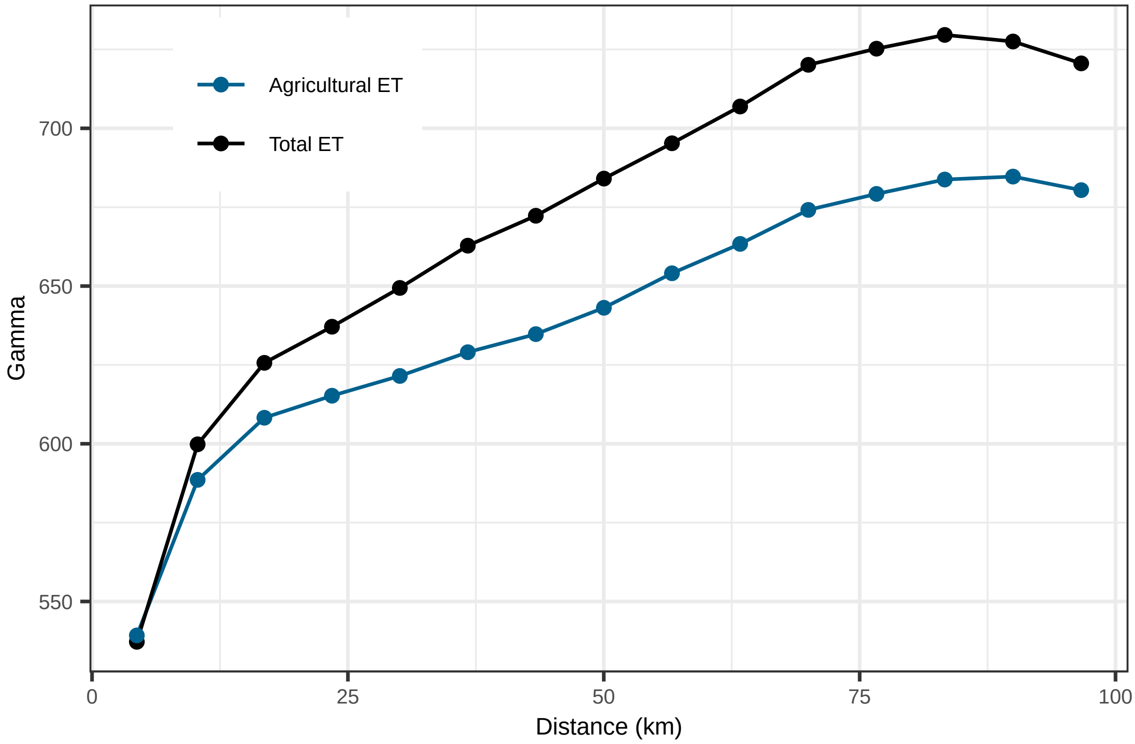

Supplementary Fig. 16 Variogram representing the spatial relationship of Total evapotranspiration (ET) and agricultural ET between nearby agricultural land. The spatial auto-correlation between pixels levels off around 75km of distance. We therefore use clustered standard errors for areas of 75km x 75km to avoid artificially low standard errors when calculating ET and agricultural ET statistics.

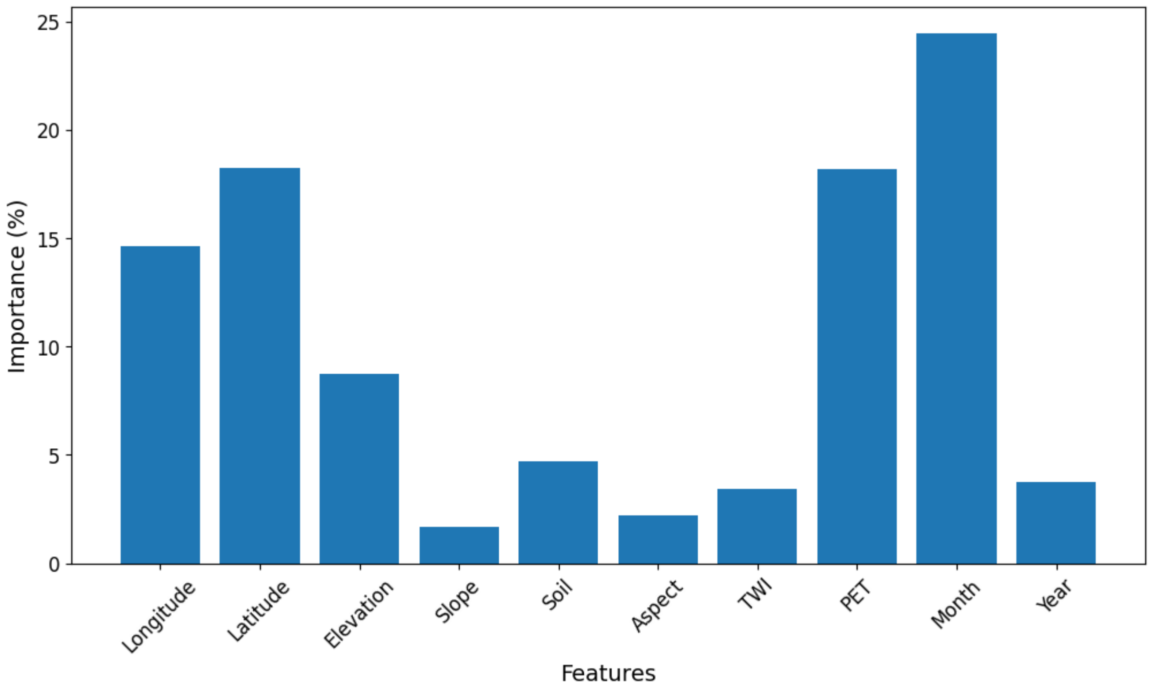

Supplementary Fig. 17 The contribution of each variable included in the gradient boosting model on its predictions of naturally-occurring ET.

Supplementary Note 7: Crop groups

The crop groups used to make Fig. 2 follow the groupings from the Department of Water Resources LandIQ dataset which we use to designate crop type in our analysis. More information on these groups can be found in the metadata for these data, and are also described here:

1. Grain and hay crops include barley, wheat, oats, miscellaneous grain and hay, and mixed grain and hay.
2. Rice includes rice and wild rice.
3. Field crops include cotton, safflower, flax, hops, sugar beets, corn (field & sweet), grain sorghum, sudan, castor beans, beans (dry), miscellaneous field, sunflowers, hybrid sorghum/sudan, millet, sugar cane, and corn.
4. Pasture includes alfalfa & alfalfa mixtures, clover, mixed pasture, native pasture, induced high water table native pasture, miscellaneous grasses, turf farms, bermuda grass, rye grass, and klein grass.
5. Truck, nursery & berry crops include artichokes, asparagus, beans (green), cole crops, carrots, celery, lettuce (all types), melons, squash, and cucumbers (all types), onions & garlic, peas, potatoes, sweet potatoes, spinach, tomatoes (processing), flowers, nursery & Christmas tree farms, mixed (four or more), miscellaneous truck, bush berries, strawberries, peppers (chili, bell, etc.), broccoli, cabbage, cauliflower, brussels sprouts, tomatoes (market), greenhouse, blueberries, Asian leafy vegetables, lettuce or leafy greens, and potato or sweet potato.
6. Deciduous fruits and nuts include apples, apricots, cherries, peaches and nectarines, pears, plums, prunes, figs, miscellaneous deciduous, mixed deciduous, almonds, walnuts, pistachios, pomegranates, and plums, prunes or apricots.
7. Citrus and subtropical include grapefruit, lemons, oranges, dates, avocados, olives, miscellaneous subtropical fruit, kiwis, jojoba, eucalyptus, and mixed subtropical fruits.
8. Vineyards include table grapes, wine grapes, and raisin grapes.

Supplementary References

[1] Orang, M. N. *et al.* California Simulation of Evapotranspiration of Applied Water and Agricultural Energy Use in California. *Journal of Integrative Agriculture* **12**, 1371–1388 (2013). URL https://www.sciencedirect.com/science/article/pii/S209531191360742X.

[2] Williams, A. P. *et al.* Observed Impacts of Anthropogenic Climate Change on Wildfire in California. *Earth's Future* **7**, 892–910 (2019). URL https://onlinelibrary.wiley.com/doi/abs/10.1029/2019EF001210. _eprint: https://onlinelibrary.wiley.com/doi/pdf/10.1029/2019EF001210.

[3] Drechsler, K., Fulton, A. & Kisekka, I. Crop coefficients and water use of young almond orchards. *Irrigation Science* **40**, 379–395 (2022). URL https://doi.org/10.1007/s00271-022-00786-y.

[4] Rohwer, J., Gerten, D. & Lucht, W. Development of functional irrigation types for improved global crop modelling. Tech. Rep. 104, Potsdam Institute for Climate Impact Research (PIK) (2007). URL http://www.pik-potsdam.de/forschung/publikationen/pik-reports/.files/pr104.pdf. Publisher: Potsdam-Institut für Klimafolgenforschung.

[5] Rost, S. *et al.* Agricultural green and blue water consumption and its influence on the global water system. *Water Resources Research* **44** (2008). URL https://onlinelibrary.wiley.com/doi/abs/10.1029/2007WR006331. _eprint: https://onlinelibrary.wiley.com/doi/pdf/10.1029/2007WR006331.

[6] Bos, M. G. & Nugteren, J. *On irrigation efficiencies* 4. ed edn. No. 19 in ILRI publication (ILRI, Wageningen, 1990).

[7] Brouwer, C., Prinks, K. & Heibloem, M. Irrigation Water Management: Irrigation Scheduling.Annex I: Irrigation efficiencies. Tech. Rep., FAO (1989). URL https://www.fao.org/3/t7202e/t7202e08.htm.

[8] Doorenbos, J. & Pruitt, W. Crop Water Requirements. FAO Irrigation and Drainage Paper 24, FAO, Rome (1977).

[9] Zhou, L. & Hoekstra, A. Y. The effect of different agricultural management practices on irrigation efficiency, water use efficiency and green and blue water footprint. *Frontiers of Agricultural Science and Engineering* **4**, 185–194 (2017). URL https://journal.hep.com.cn/fase/EN/abstract/abstract19679.shtml.

[10] Kebede, H., Fisher, D. K., Sui, R. & Reddy, K. N. Irrigation Methods and Scheduling in the Delta Region of Mississippi: Current Status and Strategies to Improve Irrigation Efficiency. *American Journal of Plant Sciences* **05**, 2917 (2014). URL http://www.scirp.org/journal/PaperInformation.aspx?PaperID=50005&#abstract. Number: 20 Publisher: Scientific Research Publishing.

[11] Bonfante, A. *et al.* LCIS DSS—An irrigation supporting system for water use efficiency improvement in precision agriculture: A maize case study. *Agricultural Systems* **176**, 102646 (2019). URL https://www.sciencedirect.com/science/article/pii/S0308521X19301465.

[12] OpenET. Intercomparison and Accuracy Report (2021).
